# Commissioning a secondary dose calculation software for a 0.35 T MR‐linac

**DOI:** 10.1002/acm2.13452

**Published:** 2022-02-15

**Authors:** Alex T. Price, Nels C. Knutson, Taeho Kim, Olga L. Green

**Affiliations:** ^1^ Department of Radiation Oncology Washington University School of Medicine St. Louis Missouri USA

**Keywords:** MRgRT, MRI‐guided radiation therapy, quality assurance, secondary dose calculations

## Abstract

Secondary external dose calculations for a 0.35 T magnetic resonance image‐guided radiation therapy (MRgRT) are needed within the radiation oncology community to follow safety standards set forth within the field. We evaluate the commercially available software, RadCalc, in its ability to accurately perform monitor unit dose calculations within a magnetic field. We also evaluate the potential effects of a 0.35 T magnetic field upon point dose calculations.

Monitor unit calculations were evaluated with (wMag) and without (noMag) a magnetic field considerations in RadCalc for the ViewRay MRIdian. The magnetic field is indirectly accounted for by using asymmetric profiles for calculation. The introduction of double‐stacked multi‐leaf collimator leaves was also included in the monitor unit calculations and a single transmission value was determined. A suite of simple and complex geometries with a variety field arrangements were calculated for each method to demonstrate the effect of the 0.35 T magnetic field on monitor unit calculations. Finally, 25 patient‐specific treatment plans were calculated using each method for comparison.

All simple geometries calculated in RadCalc were within 2% of treatment planning system (TPS) values for both methods, except for a single noMag off‐axis comparison. All complex muilt‐leaf collimator (MLC) pattern calculations were within 5%. All complex phantom geometry calculations were within 5% except for a single field within a lung phantom at a distal point. For the patient calculations, the noMag method average percentage difference was 0.09 ± 2.5% and the wMag average percentage difference was 0.08 ± 2.5%. All results were within 5% for the wMag method.

We performed monitor unit calculations for a 0.35 T MRgRT system using a commercially available secondary monitor unit dose calculation software and demonstrated minimal impact of the 0.35 T magnetic field on monitor unit dose calculations. This is the first investigation demonstrating successful calculations of dose using RadCalc in the low‐field 0.35 T ViewRay MRIdian system.

## 1 INTRODUCTION

The introduction of magnetic resonance image‐guided linear accelerators (MR‐linac) such as the ViewRay MRIdian (ViewRay, Inc., Cleveland, OH, USA) or the Elekta Unity (Elekta, Stockholm, Sweden) is a significant technologic development within the radiation oncology community.^1^, ^2^ These systems provide soft tissue contrast that is unparalleled on conventional linacs.^3^ These machines also have adaptive radiotherapy workflow tools that have enabled altered or new treatment paradigms.^4^, ^5^, ^6^, ^7^ As magnetic resonance image‐guided radiation therapy (MRgRT) continues to grow in use,^8^, ^9^, ^10^ the vendor community is developing new and modifying existing software and devices to be compatible with these MR‐linacs.^11^, ^12^, ^13^, ^14^, ^15^, ^16^, ^17^, ^18^

Software for external secondary dose calculations is one such example of a clinical tool that has been reworked by a commercial vendor to meet the needs of MRgRT technology. Secondary dose calculations are an important safety step in the clinical workflow to prevent major errors.^19^, ^20^, ^21^, ^22^ RadCalc (LifeLine Software, Austin, TX, USA) is a software that performs necessary external secondary monitor unit calculations to meet safety standards set forth within the field. Recently, RadCalc has released a software version that calculates point dose monitor unit comparisons for MRgRT, which is the only commercially available secondary dose calculation software for low‐field (ViewRay MRIdian) MRgRT at the time of this writing. RadCalc version 7.1.4.0 incorporates magnetic field effects on the beam profiles by differentiating between crossline and inline profiles. These profiles will have different profile shapes due to the Lorentz force on the secondary scattered electrons.^23^ In addition, the latest version of RadCalc characterizes the double‐stacked, double‐focused nature of the MRIdian MLCs^24^ that was otherwise not available in prior versions of RadCalc. These two additions to the RadCalc calculation algorithm are expected to improve the accuracy of the secondary monitor unit calculation.

We and others within the field have previously used in‐house methods that manipulated RadCalc to meet our clinical needs.^14^ However, using commercial software developed specifically for MRgRT secondary dose calculations provides a standardized approach across multiple clinics to meet safety standards. This is especially important considering that the commercially available MRgRT systems at the time of this writing do not provide their own external pretreatment dose calculation checks. Prior evaluations of RadCalc for a high‐field system indicated acceptable performance for point dose calculations but poor performance for plane dose comparisons within a high‐magnetic field environment.^14^ However, RadCalc's performance of secondary dose calculation comparisons with a low‐field MRgRT treatment planning system (TPS) have yet to be evaluated in the literature. In addition, there has been no formal evaluation of the impact of a 0.35 T magnetic field itself upon the accuracy/performance of monitor unit point dose calculations.

Based on these needs, we demonstrate here the commissioning and evaluation of the 0.35 T MRIdian system in RadCalc version 7.1.4.0, as well as the evaluation of a low‐field MRI on simple monitor unit calculations. The basis of this work follows the AAPM Medical Physics Practice Guideline 5.a (MPPG5a): Commissioning and QA of Treatment Planning Dose Calculations to guide both the commissioning and evaluation of the secondary dose calculation.^25^ These guidelines supply a method to verify dose calculations in a variety of scenarios applicable to magnetic field dose calculations. This work will help in guiding MRgRT users on how to successfully commission and evaluate a secondary dose calculation software for pre‐treatment secondary dose verification.

## 2 METHODS

### 2.1 Beam characterization

The ViewRay MRIdian system was modeled within RadCalc. Per manufacturer instructions, RadCalc requires the collimator scatter factor (*S*_c_), phantom scatter factor (*S*_p_), percentage depth dose curves (PDDs), and beam profiles to develop a beam model within their system.^26^ Although data are typically extracted from beam measurements, the dosimetric data for RadCalc were extracted from the ViewRay TPS (VR TPS). This was due to bore size limitations and non‐MR‐compatible measurement devices available at the time of commissioning which limits full beam characterization needed for RadCalc. Each field size less than 5 × 5 cm^2^ was calculated using the Monte Carlo engine of the TPS with the magnetic field on, 0.1 × 0.1 × 0.1 cm^3^ dose grid, and 0.2% uncertainty. For fields greater than 5 × 5 cm^2^, the uncertainty was changed to 0.5% to increase calculation speed. Once calculated, dose plane files were exported and post‐processed in MATLAB (MathWorks, Natick, MA, USA), extracting the PDDs and profiles wanted while applying a smoothing filter to the data. The data were then imported into RadCalc. The square field sizes used for the output factors, PDDs, and profiles were 1.66 × 1.66, 2.49 × 2.49, 3.32 × 3.32, 4.98 × 4.98, 5.81 × 5.81, 7.47 × 7.47, 9.96 × 9.96, 14.94 × 14.94, and 19.92 × 19.92 cm^2^. For the profiles, depths of 1.4, 5, 10, and 20 cm were included. The inclusion of distinguishing which profile is inline or crossline allows RadCalc to use both profiles in the monitor unit calculation. Each are subject to magnetic field effects, especially the crossline, due to the Lorentz force.

For the *S*_c_ and total scatter factor (*S*_cp_) calculations, conditions set for the simulation were 80 cm source to surface distance (SSD) at a 10 cm depth. Dose points were extracted at this location for all *S*_cp_ calculations in a uniform water‐equivalent cubic digital phantom. For the *S*_c_ simulation, the setup was done in air with a water‐equivalent build‐up cap for fields larger than 4.98 × 4.98 cm^2^. For smaller fields, a brass build‐up cap (1 cm diameter, 1.3 cm in length) was simulated in the TPS and calculated based on the recommendations in TG‐74.^27^

For the calculation methods, RadCalc performs monitor unit point dose calculations for all other non‐MRgRT linacs with a single profile that does not differentiate between inline and crossline profiles. This method will be known as the noMag method in this study. The noMag method used the crossline profile that is subject to the Lorentz force as the profile for all calculations, that is, both the inline and crossline calculations. Conversely, in the most recent version of RadCalc, users can differentiate between inline and crossline profiles, which are both impacted differently by the 0.35 T magnetic field. This results in differing off‐axis (OAX) ratios depending on the location of the calculation point. This method will be called the wMag method for the remainder of this study.

**FIGURE 1:**
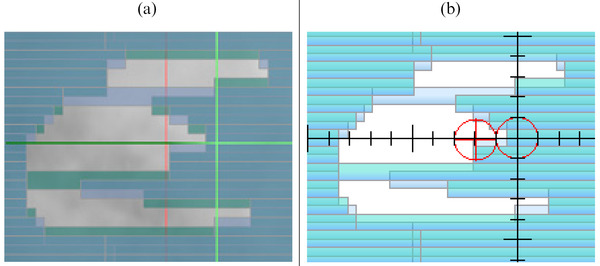
(a) MRIdian MLCs that only have one single leaf in the middle of the field. This single leaf would have a transmission value of approximately 35.0%. A closed set of interdigitated single layered MLCs has a transmission value of approximately 7.8%. All of the other leaves have mostly double‐stacked MLC patterns which have a transmission of approximately <0.1%. (b) The MLCs as modeled in RadCalc version 7.1.4.0

Both methodologies model the double‐stacked MLCs, which was not available in prior versions of RadCalc. Of note, RadCalc accepts only a single MLC transmission value in all scenarios of field blocking. However, there are many scenarios where there is a single leaf within the treatment field, extending past the double‐stacked MLCs. Figure 1 illustrates this scenario. This creates uncertainty in selecting a uniform MLC transmission value. To account for the differing MLC transmission values between single and double‐stacked MLCs within the treatment field, an approximate MLC transmission value for a combination of both scenarios was chosen. To decide this transmission value, intensity modulated radiation therapy (IMRT) RadCalc results were calculated with varying MLC transmission percentages of 1.5%, 1.625%, and 1.75%. The transmission value that produced the best overall average patient calculation results was chosen for all subsequent calculations. These transmission values were chosen based on the approximate transmission value for the ViewRay MR‐Cobalt system reported by Cai et al.^28^ and our institutional commissioning results.

### 2.2 Simple geometries and field arrangements

Once the machine was characterized within RadCalc, a suite of simple geometries and field arrangement calculations were evaluated. The calculations described below were performed for both the noMag and wMag methods. The phantom used for these calculations was a uniform water‐equivalent cubic digital phantom.

Due to the MLC configuration on the ViewRay MRIdian, reference conditions are defined for a 9.96 × 9.96 cm^2^ field at 80 cm SSD and a 10 cm depth for a 6‐MV flattening filter‐free (FFF) beam. To test the system's ability to calculate dose in another set of reference conditions, TG‐51 calibration condition fields were calculated in RadCalc and compared to the expected dose of 100 cGy when delivering 100 MU at depth of dose maximum (the institutional reference condition).

Large field (19.92 × 19.92 cm^2^) calculation accuracy was verified in RadCalc by comparing against the VR TPS calculation using the settings of 0.1 × 0.1 × 0.1 cm^3^ dose grid, magnetic field on, and 0.2% uncertainty. OAX calculations were performed at a variety of depths and an OAX distance of 7.5 cm in both the X and Y directions. Calculations were performed for SSDs of 70, 80, and 90 cm to investigate the correlation of different SSDs with secondary dose calculation accuracy.

Small field (2.48 × 2.48 cm^2^) RadCalc calculation accuracy was evaluated by comparing against VR TPS calculations using a setting of 0.1 × 0.1 × 0.1 cm^3^ dose grid, magnetic field on, and 0.2% uncertainty. OAX calculations were performed at 5 and 20 cm depths and were ±0.5 cm OAX in the X and Y directions. SSD for the small field calculations was 80 cm.

**FIGURE 2:**
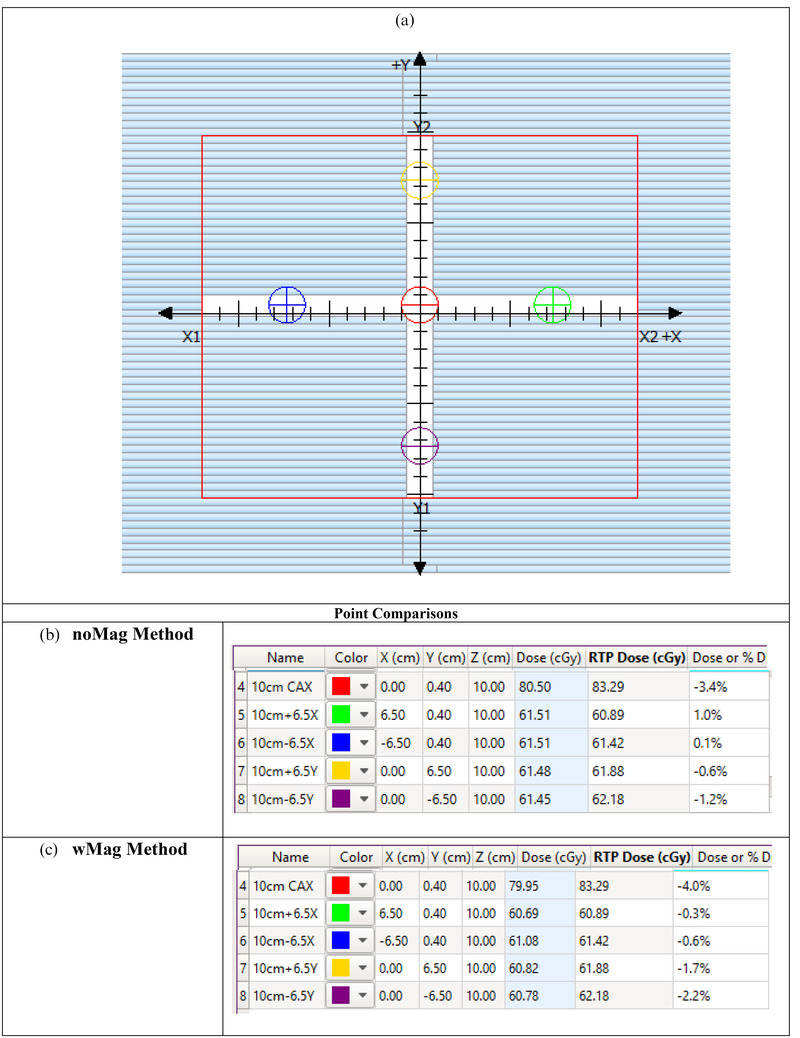
(a) The cross‐pattern used to test complex MLC patterns. The calculation points identified in (a) have corresponding calculation results in (b and c). The calculated doses and percentage differences are presented. (b) The noMag method and (c) the wMag method. In the name column, the first value for the description is the depth whereas the second value is the distance in cm off‐axis

A small complex MLC field (1.5 cm width between the X‐MLCs and 1.25 cm between parallel Y‐MLCs) in the shape of a cross‐pattern (Figure 2a) was compared in RadCalc against VR TPS calculation with the settings of 0.1 × 0.1 × 0.1 cm^3^ dose grid, magnetic field on, and 0.25% uncertainty. A dose calculation point was placed in the center of each “spoke.” All points were at a 10 cm depth.

### 2.3 Complex geometries and investigation of magnetic field effects

**FIGURE 3:**
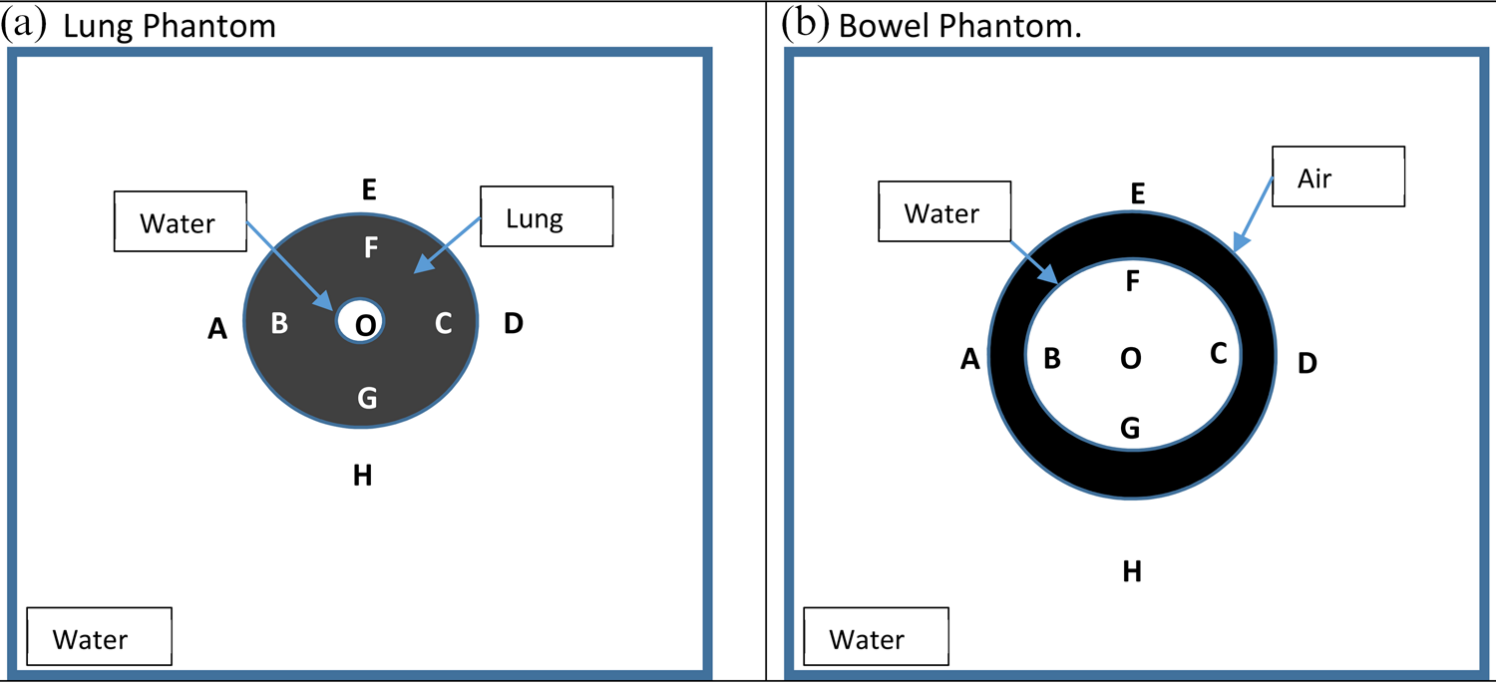
Complex heterogeneous phantoms used for calculation. (a) The lung phantom and (b) the bowel phantom

Once simple geometries were investigated, more complex scenarios were evaluated to determine the limits of the system. Two separate, complex phantom geometries were used to investigate the impact of heterogeneity on simple point dose monitor unit calculations in the presence of the magnetic field. The first geometry (lung phantom) represents a lesion within a lung volume. The second phantom contains a torus of air surrounding a larger water tissue area. This phantom represents an area of bowel gas (bowel phantom). These phantoms are illustrated in Figure 3.

In both the lung and bowel phantoms, the larger volume was a uniform water‐equivalent cubic digital phantom (1 g/cc). In the lung phantom, the lung volume (0.26 g/cc) was 10 cm in diameter with a 2.5 cm water target in the middle. In the bowel phantom, the major radius of the torus was 6 cm and the minor radius was 2 cm in length. The torus was filled with air (0.001 g/cc). In each phantom, percentage differences between the ViewRay TPS and RadCalc dose calculation points were compared at each location represented by the letters in Figure 3. These locations were roughly 2 cm from a heterogeneity. For both the lung and bowel phantoms, an AP field, a set of lateral fields, and a four‐field box arrangement using a field size of 4.98 × 4.98 cm^2^ were calculated. Additionally, to characterize the impact of the electron return effect near air/tissue interfaces, comparison points 1 cm from the air torus in the bowel phantom were chosen for evaluation. This is the minimum distance that a calculation comparison point should be from a heterogeneity as suggested per TG‐71.^26^ This comparison was only done for the four‐field box arrangement.

To further demonstrate the isolated effects of a 0.35 T magnetic field on point dose monitor unit calculations, a 2.5 × 2.5 cm^2^ AP, set of lateral fields, and a four‐field box field arrangement was delivered to a uniform water‐equivalent cubic digital phantom. A small field was chosen because the effect of the dose deposition from the magnetic field is greater at smaller fields.^23^ Percentage differences between RadCalc and the ViewRay TPS calculation were evaluated at the isocenter (15 cm depth), and 0.5 cm OAX in both the positive and negative *X*–*Y* directions. A separate set of four additional 2.5 × 2.5 cm^2^ fields were calculated 6.25 cm OAX in the positive and negative *X*–*Y* directions to test RadCalc's ability to calculate for an isolated OAX field.

### 2.4 Evaluation of clinical plans

Finally, to evaluate performance in complex clinical treatment plans, 25 anonymized patient plans for several sites were evaluated in RadCalc and compared against the VR TPS. The list of patient treatment sites, modality, and fractionation schedule are shown in Table 1. For the IMRT plans, step‐and‐shoot MLC (sMLC) delivery was delivered. The location of points was chosen to be placed at the centroid of the planning planning target volume (PTV). This provided a consistent method of point placement across all patients and would not bias the results for the “best” point. Calculations in the ViewRay TPS were done with a 0.3 × 0.3 × 0.3 cm^3^ dose grid, magnetic field on, and the uncertainty equal to 0.5%. All comparisons were done in a composite dose mode. ViewRay does not provide per beam dose point information. Both the noMag and wMag methods were performed without the ROI module unless otherwise noted. All parameters relied on the effective path length extracted from the ViewRay plan overview file. A description of how RadCalc reads the plan overview file is in the supplementary data. Statistical significance was calculated for all differences in patient calculations using a two‐tailed paired *t*‐test with significance defined at *p* < 0.05 in Microsoft Excel (Microsoft, Redmond, WA, USA).

**TABLE 1:** Disease site, modality, fractionation scheme, and comparison of the RadCalc percentage difference of the ViewRay plan dose and the version 7.1.4.0 calculated dose

Methods of verifying calculated dose			
Site	Modality	Fractionation scheme	RadCalc % difference
Abdomen	SBRT	700 cGy × 5	2.4%
**Abdomen** **^a^**	SBRT	1000 cGy × 5	3.8% (**6.1%** **^a^**)
Breast	SBRT	850 cGy × 3	2.1%
Breast	SBRT	850 cGy × 3	–0.7%
Chestwall	IMRT	200 cGy × 30	–4.2%
Chestwall	SBRT	700 cGy × 5	1.2%
Liver	SBRT	1000 cGy × 5	–0.6%
Liver	SBRT	1000 cGy × 5	–0.5%
Liver	SBRT	1000 cGy × 5	–1.1%
Liver	SBRT	1000 cGy × 5	3.9%
Liver	SBRT	1000 cGy × 5	–2.6%
Liver	SBRT	1000 cGy × 5	4.0%
Liver	SBRT	1000 cGy × 5	0.4%
Lung	IMRT	500 cGy × 12	–1.0%
**Lung** **^a^**	IMRT	400 cGy × 15	–0.3% (**7.6%** **^a^**)
**Lung** **^a^**	IMRT	400 cGy × 15	–2.5% (**11.2%** **^a^**)
Pancreas	SBRT	1000 cGy × 5	1.1%
Pancreas	SBRT	1000 cGy × 5	–3.1%
Pancreas	SBRT	1000 cGy × 5	3.0%
Pancreas	SBRT	1000 cGy × 5	–1.2%
Pancreas	SBRT	1000 cGy × 5	2.7%
Pancreas	SBRT	1000 cGy × 5	2.9%
**Pancreas** **^a^**	SBRT	1000 cGy × 5	–3.8% (**5.1%** **^a^**)
Pancreas	SBRT	1000 cGy × 5	–4.3%
Periportal	SBRT	700 cGy × 5	0.5%

^a^ Bold treatment site represents significant heterogeneity within the beam path.

Abbreviation: SBRT, Stereotactic body radiation thearpy.

## 3 RESULTS

### 3.1 Beam characterization

**FIGURE 4:**
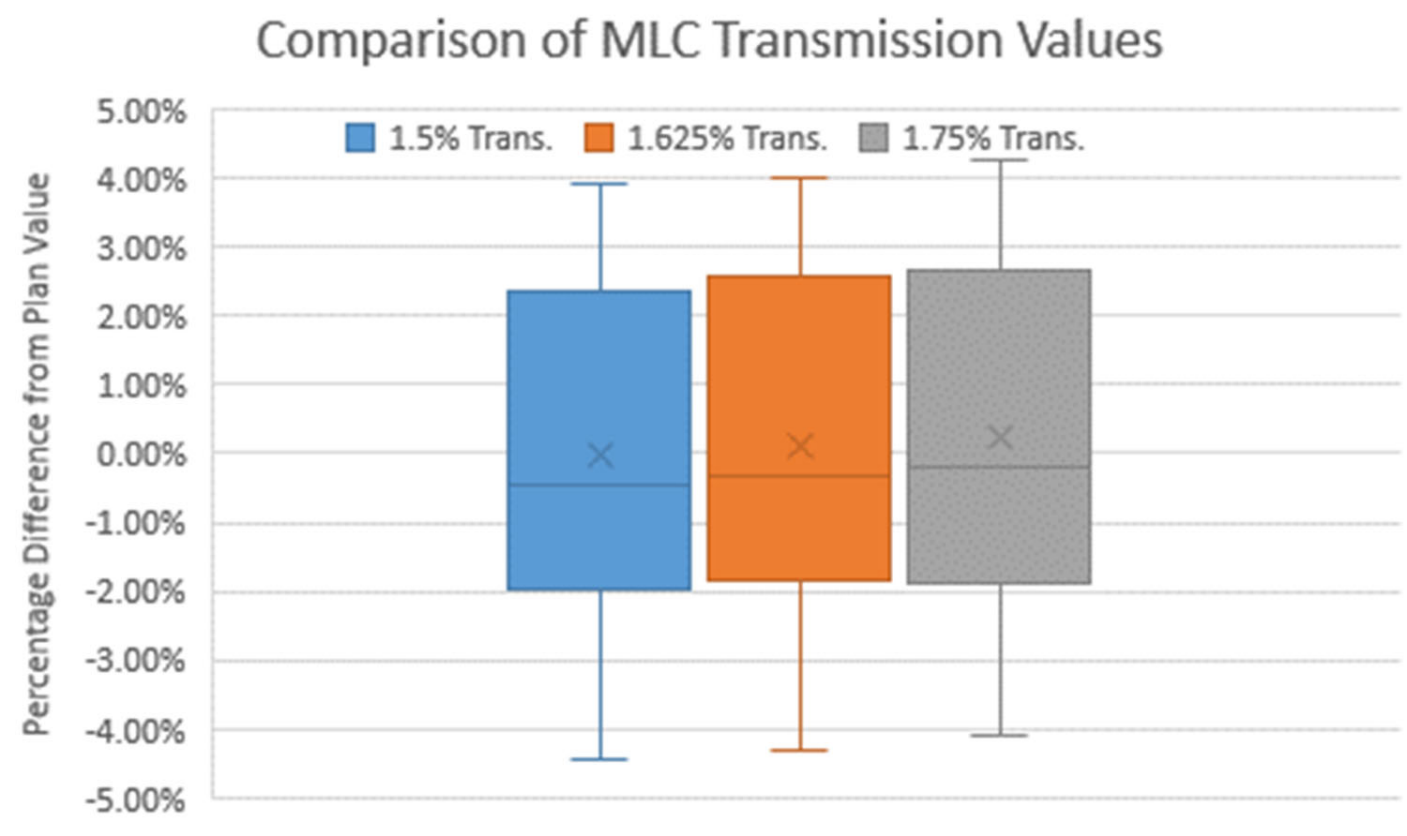
Box and whisker plot of the 25 patient results with varying levels of transmission values. Note that 1.5% transmission is the data on the left, 1.625% MLC transmission is in the middle, and 1.75% MLC transmission is on the right

Data extracted from ViewRay TPS and manipulated in MATLAB were successfully imported into the physics beam characterization module within RadCalc. An MLC transmission value of 1.625% resulted in the best average patient‐specific plan results after iterating through a set of transmission values. The distribution of results between 1.5%, 1.625%, and 1.75% are shown in Figure 4. The radiation/light field offset was set to 0.0 cm due to the double‐focused nature of the MLCs.

### 3.2 Simple geometries

For the reference TG‐51 geometry, both the noMag and wMag methods were –0.02% lower than what was expected. For the large field calculations, both methods were within 2%. For the noMag method, the average result was 0.1 ± 0.7% and the median was 0.1%. For the wMag method, the average result was –0.3 ± 0.9% and the median was 0.0%. These are all within 2%: MPPG5a tolerance. The large field results are shown in Table 2.

**TABLE 2:** Comparison of the noMag and wMag methods for large fields and changing source to surface distance (SSD) scenarios

Large field comparison						
	noMag method			wMag method		
Location	70SSD	80SSD	90SSD	70SSD	80SSD	90SSD
*d* = 20 cm, CAX	0.5%	0.4%	0.5%	0.5%	0.4%	–0.5%
*d* = 25 cm, +7.5 cm X‐OAX	–0.2%	0.7%	1.3%	0.0%	0.7%	0.9%
*d* = 25 cm, ‐7.5 cm X‐OAX	–1.3%	–0.6%	0.3%	–1.7%	–0.8%	0.3%
*d* = 10 cm, +7.5 cm Y‐OAX	0.0%	–0.1%	0.9%	–1.0%	–0.9%	0.4%
*d* = 10 cm, ‐7.5 cm Y‐OAX	0.9%	–0.6%	0.1%	–1.9%	–1.4%	–0.5%

*Note*: All points are within 2% which is within MPPG5a tolerances.

Abbreviations: MPPG5a, Medical Physics Practice Guideline 5.a; OAX, off‐axis.

Moving towards smaller fields, results were ≤2.0% for both methods except for the noMag method in the positive crossline direction. For the noMag method, the average result was 1.1 ± 0.7% and the median was 0.9%. For the wMag method, the average result was –0.9 ± 0.4% and the median was 0.8%. The small field results are shown in Table 3. The small field MLC geometry shown in Figure 2 had results of <5% for both methods.

**TABLE 3:** Comparison of the noMag and wMag methods for small fields

Small field comparison		
Location	noMag method	wMag method
*d* = 10 cm, CAX	0.9%	0.5%
*d* = 20 cm, +0.5 cm X‐OAX	2.2%	1.6%
*d* = 20 cm, ‐0.5 cm X‐OAX	0.3%	0.8%
*d* = 5 cm, +0.5 cm Y‐OAX	1.4%	1.1%
*d* = 5 cm, ‐0.5 cm Y‐OAX	0.7%	0.4%

*Note*: All points are within 2% which is within MPPG5a tolerances except for single OAX noMag comparison.

Abbreviations: MPPG5a, Medical Physics Practice Guideline 5.a; OAX, off‐axis.

### 3.3 Complex geometries and investigation of magnetic field effects

For the lung and bowel geometries, the calculation comparisons are presented in Table 4. For the lung phantom geometry, all calculations were within 5% except for the AP field in both methods. For the bowel phantom geometry, all calculation comparisons were also within 5%. For both complex geometries, the noMag method had an average result of –0.1 ± 2.9% and the median was 0.6%. For the calculations done with the wMag method, the average result was 0.4 ± 2.7% and the median was 1.0%. Additionally, calculation comparison points that were 1 cm from the gas heterogeneity were also within 5%. These are shown in Table 5.

**TABLE 4:** Comparison of the noMag and wMag methods for variety of field arrangements for both the lung and bowel phantom

Heterogeneity comparison												
	noMag method						wMag method					
Location	AP‐L	2Fd‐L	4Fd‐L	AP‐B	2Fd‐B	4Fd‐B	AP‐L	2Fd‐L	4Fd‐L	AP‐B	2Fd‐B	4Fd‐B
O	3.96%	3.98%	4.76%	1.67%	1.48%	1.03%	4.18%	3.96%	4.76%	1.65%	1.48%	0.87%
A	N/A	–3.47%	–2.86%	N/A	0.94%	0.60%	N/A	–3.40%	–2.36%	N/A	1.04%	1.05%
B	N/A	N/A	N/A	N/A	1.80%	–2.86%	N/A	N/A	N/A	N/A	1.87%	–0.17%
C	N/A	N/A	N/A	N/A	1.12%	–2.82%	N/A	N/A	N/A	N/A	1.16%	–0.09%
D	N/A	–3.04%	–2.42%	N/A	0.60%	0.33%	N/A	–3.04%	–1.97%	N/A	0.64%	0.76%
E	0.03%	N/A	–2.82%	0.58%	N/A	3.24%	0.15%	N/A	–2.16%	0.58%	N/A	3.63%
F	N/A	N/A	N/A	1.27%	N/A	–2.04%	N/A	N/A	N/A	1.26%	N/A	0.36%
G	N/A	N/A	N/A	3.23%	N/A	–0.60%	N/A	N/A	N/A	3.25%	N/A	1.87%
H	–8.47%	N/A	–4.87%	1.69%	N/A	1.25%	–8.46%	N/A	–4.27%	1.67%	N/A	1.64%

*Note*: “‐L” denotes the lung phantom, whereas the “‐B” denotes the bowel phantom.

**TABLE 5:** Comparison of the noMag and wMag methods for calculation points at 1 cm from the gas heterogeneity in the bowel phantom

Near heterogeneity comparison		
Location	noMag method	wMag method
A	2.65%	2.79%
B	3.33%	3.43%
C	4.03%	4.15%
D	1.70%	1.82%

For the 2.5 × 2.5 cm^2^ AP field, the average point comparison percentage differences between the ViewRay TPS and RadCalc were 1.5% and 1.3% for the noMag and wMag methods, respectively. For the AP/PA field, the average percentage differences were 1.2% and 0.9% for the noMag and wMag methods, respectively. When adding more fields to create a four‐field box arrangement, the average percentage difference was –0.1% for the noMag and wMag methods.

For the isolated OAX calculations in the negative *X* direction, the noMag and wMag methods percentage differences were 1.0% and 1.1%, respectively. In the positive *X* direction, the percentage differences were 1.5% and 1.4% for the noMag and wMag methods, respectively. For the isolated OAX calculations in the negative Y direction, the noMag and wMag methods percentage differences were 1.8% and 2.0%, respectively. In the positive *Y* direction, the percentage differences were 1.2% and 1.4% for the noMag and wMag methods, respectively.

### 3.4 Evaluation of clinical plans

**FIGURE 5:**
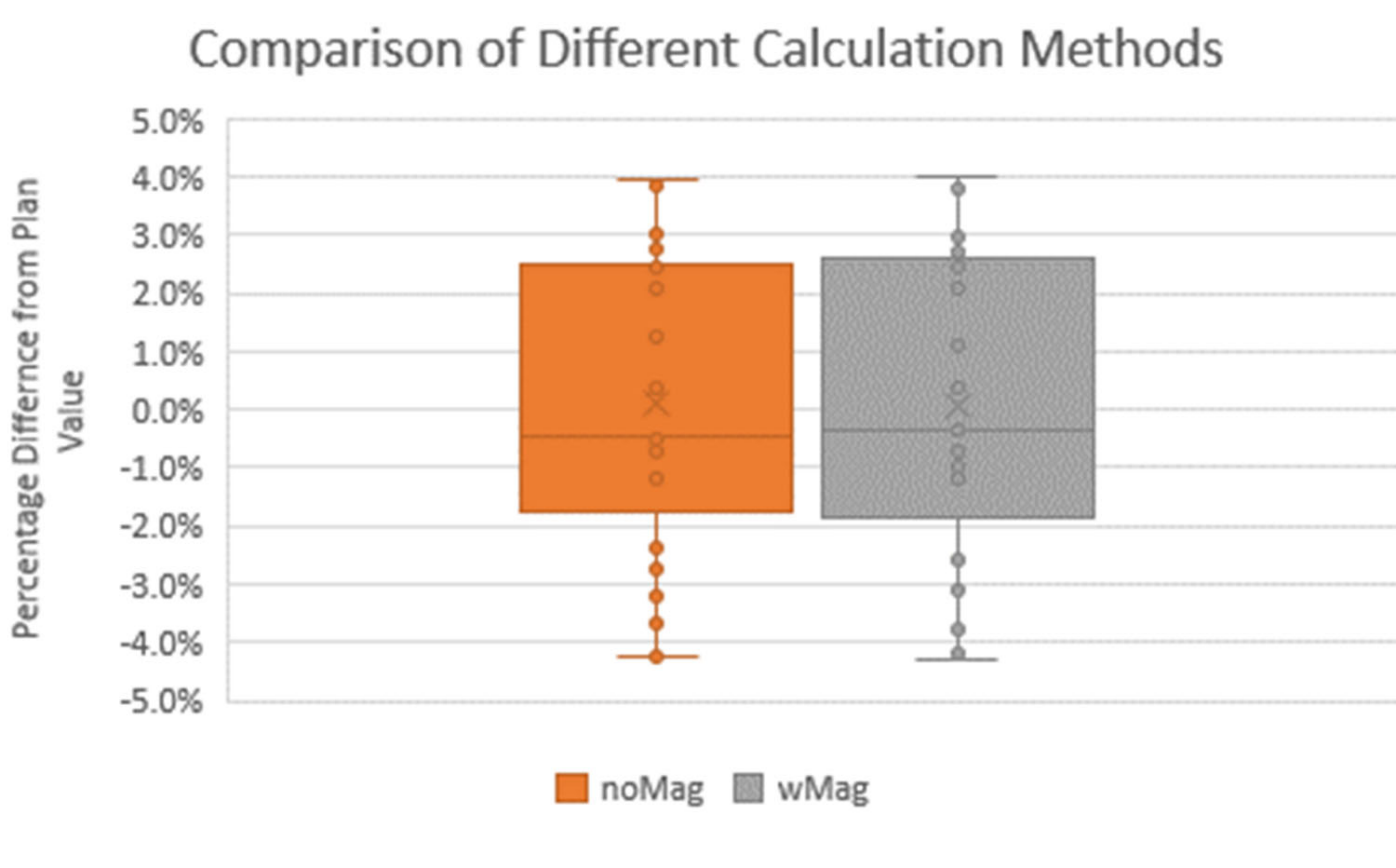
Box and whisker plot of the 25 patient results with using different calculation methods

For both the noMag and wMag methods, all 25 patients were successfully calculated and within the 5% tolerance. The average results for the noMag and wMag methods were 0.09 ± 2.5% and 0.08 ± 2.6%, respectively. The results are shown in Figure 5. There was no statistically significant difference between the noMag and wMag methods, *p* = 0.7. In both the noMag and wMag methods, only four of the 25 needed calculation adjustment including effective path length with field size correction or the ROI method correction for some density scenarios.^29^ All results are shown in Table 1.

## 4 DISCUSSION

We have successfully commissioned and evaluated an MRgRT point dose monitor unit secondary dose calculation software for the ViewRay MRIdian system to within accepted practice tolerances. Our method tests the various levels of details that can be introduced into a secondary monitor unit calculation software. This includes investigating the transmission value for the double‐stacked MLC. It also includes investigating the magnetic effects on profiles used for calculation. Performing external secondary dose calculations provides a necessary safety measure in the radiation oncology workflow process. All simple geometries and patient‐specific calculations in the secondary dose calculation software compared favorably with the TPS calculation.

The effect of a 0.35 T magnetic field has historically been inadequately described within the radiation oncology literature for secondary point dose monitor unit calculations. The Lorentz force caused by the magnetic field, mostly in the crossline direction, would be the greatest anticipated challenge to a monitor unit calculation. However, RadCalc version 7.1.4.0 can indicate which direction the Lorentz force is being applied and incorporates the differences in beam profiles within the calculations. The large field calculations in RadCalc when compared with the calculations performed in the ViewRay TPS were within MPPG5a tolerances. Similar results were seen when using both small fields and more complex MLC geometries which have more pronounced magnetic field effects in the beam profile shape. This was also the case when investigating small fields OAX. Thus, for simplistic and complex geometries within a uniform water bath, the monitor unit point dose calculations are within acceptable community standards, indicating that RadCalc can appropriately calculate the magnetic field impact with either method (noMag vs. wMag).

In the complex heterogeneous phantom geometries, there were again minimal differences between the calculations performed in RadCalc and the ViewRay TPS. In the bowel phantom, all calculation points were within the MPPG5a standard of 5% which is especially important considering that the majority of our patient load are abdominal treatment sites. Even in the more challenging lung phantom, all calculation points were within tolerance except the distal point in the lung phantom for a single AP field. This scenario had a greater loss of secondary electrons from the influence of the Lorentz force and is difficult to explicitly account for within RadCalc. However, when more beams are added to the calculation, the Lorentz force skewed profile largely cancels out when beams are opposing. This has also been shown by other investigators.^30^, ^31^ At our institution, we consistently use over 20 beams for IMRT, resulting in the Lorentz force impact being largely mitigated in our patient‐specific plan calculations. In summary, monitor unit calculations will potentially struggle with single field isolated calculation points in a heterogeneous medium or calculation points less than 1 cm from the lung/gas tissue interface, due to a greater impact by the magnetic field on the dose deposition.^32^, ^33^ This is currently a limitation of the software and other non‐Monte‐Carlo dose calculation software. Nonetheless, this was largely mitigated when using multiple fields in clinical treatments.

For patient‐specific calculations, placing the RadCalc point in the middle of the planning PTV was a necessary step to limit the bias of selecting the “best” point for comparison to influence the results. This could have resulted in points placed on a gradient such as a C‐shaped target. However, the majority of generic point placements still produced results within MPPG5a guidelines which was also seen in the high‐field MR environment.^14^ It should be noted that four plans needed common monitor unit calculation manipulations to be within MPPG5a guidelines. Three plans needed the field size correction due to loss of lateral scatter contributions from the low‐density lung or bowel gas regions adjacent to the target/calculation point. The fourth plan (lung plan with 11.2% initial difference) needed an ROI density correction for both the oblique nature of the beams impinging on the patient's surface and lung density surrounding the target. These adjustments would have been needed for any treatment planning scenario regardless of the presence of a magnetic field.

When comparing the noMag and wMag methods in simple and complex geometries, the noMag and wMag methods were similar to each other with minor, clinically acceptable variation. Patient calculations percentage differences for both the noMag and wMag methods were centered about <0.1% for the patient calculations with non‐significance between the two. When comparing crossline and inline profiles, the differences across each profile within a water bath are not prominent enough to be discerned in patient calculations. This leads us to the conclusion that the inclusion of the magnetic field in both profile directions has minimal impact on the monitor unit calculation accuracy for 0.35 T MRgRT. This is even the case of OAX calculation points. However, we do feel it is important to include the profile that has the magnetic field effects to account for OAX calculation points. This could potentially have an impact on palliative clinical setup scenarios where simple calculations are used for treatment. However, most clinical setup palliative treatments are treated on central axis, thus limiting any differences for any OAX calculations.

One critique of our study could be that we used TPS‐calculated data in RadCalc, which supplies cleaner and more complete data for machine characterization in RadCalc. We assume this to be like using pre‐configured data such as other widely used TPSs.^34^, ^35^, ^36^ This allows us to demonstrate the feasibility and process of commissioning a point dose monitor unit calculation software, albeit we do lose some separate independence as a secondary dose calculation check. We were limited in our ability to input our measured beam data into RadCalc due to bore size limitations and measuring device limitations on MRgRT systems, such as non‐MR‐compatible water tanks. This challenge is shared across current MRgRT platforms due to inherent challenges of measurements in an MRI bore.^14^ PDDs would not be deep enough for RadCalc and the profiles are captured with an array device or raster scanning a 1D water tank by moving the couch in a piecewise fashion.^35^, ^37^ With that said, our measured data at the time of commissioning were compared to the closed‐system pre‐configured TPS. The TPS's closed‐system Monte‐Carlo phase space model was adjusted to better match our beam data via multiple iterations. All subsequent commissioning measurements taken matched favorably with our calculated data and are not the topic of this study. Finally, we do encourage all users with MR‐compatible measurement devices to compare their data as best as possible with ViewRay TPS data during the commissioning process.^34^ Novel MR‐compatible are in development that will subsequently improve measuring capabilities on multiple MRgRT platforms.^11^

In our investigation, we have successfully shown that simple point dose monitor unit calculations can be used for most situations in a 0.35 T MR‐linac. To date, this is the first study that successfully calculates monitor unit calculations using profiles with the magnetic field effects incorporated in a low‐field environment. Using these techniques, dose distributions for a multitude of treatment techniques and anatomical sites can be checked with independent point dose monitor unit calculations.

## 5 CONCLUSION

In our study, we were able to commission and evaluate a commercial monitor unit secondary dose calculation software for the ViewRay MRIdian system. We performed calculations in a variety of phantom geometries and a variety of patient‐specific plan comparisons with satisfactory results. The ability to perform MRgRT secondary dose calculations provides the necessary safety measures needed within the radiation oncology workflow.

## CONFLICT OF INTEREST

Dr. Green and Mr. Price report personal fees from ViewRay, Inc., outside the submitted work. Mr. Price also reports personal fees from SunNuclear Corporation outside the submitted work.

## AUTHOR CONTRIBUTIONS

Mr. Price and Dr. Green were involved in the conceptualization, investigation, and methodology. Mr. Price is responsible for the writing of this manuscript and data collection. Dr. Kim aided in data collection. Dr. Knutson provided expertise from prior secondary dose calculation research.

## ETHICS STATEMENT

All patients included in this study were treated with MRgRT in 2020 and are part of a prospective MRIgRT patient registry (registry #2013111222).

## Supporting information



SUPPORTING INFORMATION
