# Supplementary material for: Commissioning a secondary dose calculation software for a 0.35 T MR‐linac
Source: J Appl Clin Med Phys. 2022 Feb 15;23(3):e13452. doi: 10.1002/acm2.13452 (PMC8906210; doi:10.1002/acm2.13452)
Supplement: Supplementary file 1 — SUPPORTING INFORMATION [file ACM2-23-e13452-s001.docx]

2.2 DICOM Converter

The ViewRay treatment planning system exports data in a format that does not follow standard DICOM convention for the MLCs and point doses. Thus, DICOM manipulation was needed to correctly import the data into RadCalc. An in-house MATLAB code was written to accomplish this task. The code requires a DICOM RT-Plan file, DICOM RT-Dose point file, and the plan overview text file produced by the VR TPS. RadCalc Version 7.1.4.0 requires the same data and performs similar operations as the in-house DICOM manipulation code.

The code first reads in the plan overview text file, extracting gantry angles, segments per beam, MU and weight per segment, and MLC position per segment. Once extracted, the code re-writes the DICOM RT-Plan file, updating the MLC positions, adding jaws to match the maximum extent of the MLCs, and updating the weighting value for each segment. Version 7.1.4.0 is also able to extract depths and effective depths per field.

The global dose value was then inserted into the DICOM file for dose tracking. Since ViewRay does not export a dose per field value, it is not possible to determine what an appropriate dose per field value would be without significant and time-consuming plan manipulation.
